# Behaviour and muscle activity across the aquatic–terrestrial transition in *Polypterus senegalus*

**DOI:** 10.1242/jeb.243902

**Published:** 2022-12-09

**Authors:** Keegan Lutek, Kathleen L. Foster, Emily M. Standen

**Affiliations:** ^1^ University of Ottawa, Department of Biology, 30 Marie-Curie Private, Ottawa, ON, Canada, K1N 9A7; ^2^ Ball State University, Department of Biology, 1600 Ashland Avenue, Muncie, IN 47306, USA

**Keywords:** Amphibious fishes, Kinematics, Electromyography, Terrestrial locomotion, *Polypterus*, Locomotor transitions

## Abstract

Amphibious fishes moving from water to land experience continuous changes in environmental forces. How these subtle changes impact behavioural transitions cannot be resolved by comparisons of aquatic and terrestrial locomotion. For example, aquatic and terrestrial locomotion appear distinct in the actinopterygian fish *Polypterus senegalus*; however, it is unclear how gradual water level changes influence the transition between these locomotor behaviours. We tested the hypothesis in *P. senegalus* that swimming and walking are part of an incremental continuum of behaviour and muscle activity across the environmental transition from water to land rather than two discrete behaviours, as proposed by previous literature. We exposed *P. senegalus* to discrete environments from fully aquatic to fully terrestrial while recording body and pectoral fin kinematics and muscle activity. Anterior axial red muscle effort increases as water depth decreases; however, a typical swimming-like anterior-to-posterior wave of axial red muscle activity is always present, even during terrestrial locomotion, indicating gradual motor control changes. Thus, walking appears to be based on swimming-like axial muscle activity whereas kinematic differences between swimming and walking appear to be due to mechanical constraints. A discrete change in left–right pectoral fin coordination from in-phase to out-of-phase at 0.7 body depths relies on adductor muscle activity with a similar duty factor and adductor muscle effort that increases gradually as water depth decreases. Thus, despite distinct changes in kinematic timing, neuromuscular patterning is similar across the water depth continuum. As the observed, gradual increases in axial muscle effort reflect muscle activity changes between aquatic and terrestrial environments observed in other elongate fishes, a modified, swimming-like axial muscle activity pattern for terrestrial locomotion may be common among elongate amphibious fishes.

## Teaser

**Highlighted Article:** Despite discrete changes in kinematic magnitude and coordination, the pattern of axial red muscle and pectoral fin adductor activity remains similar across the aquatic–terrestrial transition in *Polypterus senegalus*.

## INTRODUCTION

Amphibious fishes move through disparate physical environments using a single locomotor control system (central pattern generators, musculoskeletal elements and sensory systems). The locomotor control system in fishes is adapted for an aquatic environment. To facilitate terrestrial movement, some species have evolved specialized structures (e.g. pectoral fins in mudskippers; Harris, 1960; Murdy, 1989) while others rely only on the locomotor flexibility of unspecialized morphology (e.g. *Polypterus senegalus* and *Kryptolebias marmoratus*; Foster et al., 2018; Perlman and Ashley-Ross, 2016; Pronko et al., 2013; Standen et al., 2014; Standen et al., 2016).

**Fig. 1.**
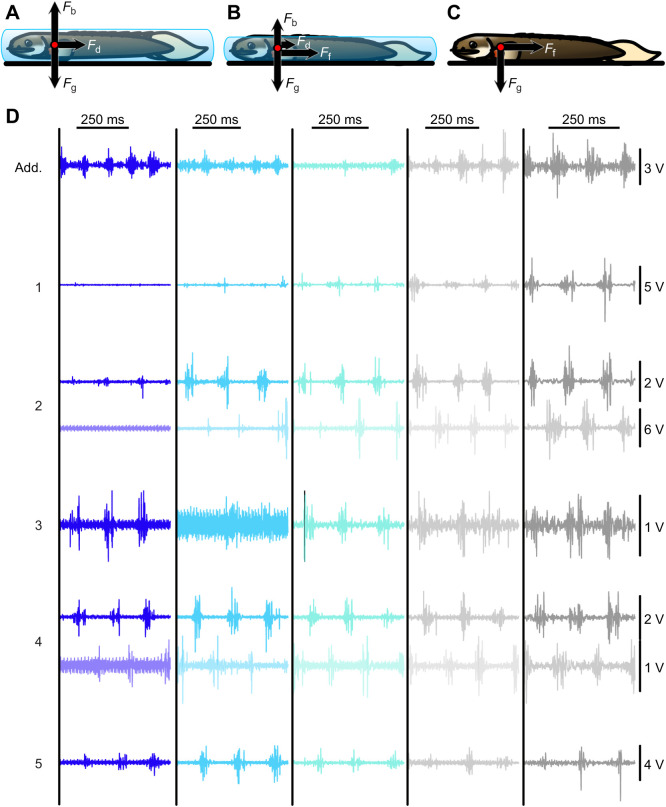
**Physical forces and example electromyography (EMG) traces for *Polypterus senegalus* across the aquatic–terrestrial transition.** Free body diagram for *P. senegalus* in water depths of (A) 1.1 body depth (BD), (B) 0.9 BD and (C) 0.0 BD. For simplicity, we assume the centre of buoyancy and centre of gravity are in the same position on the fish and all force vectors are drawn as acting at this point (red circle). Arrow size represents force magnitude, but these magnitudes are not scaled. Thus, differences in arrow size are qualitative, not quantitative. *F*_b_, buoyant force; *F*_d_, drag force; *F*_g_, gravitational force; *F*_f_, frictional force. (D) Example EMG traces for a *P. senegalus* moving in water depths of (from left to right): 3.0, 1.1, 1.0, 0.9 and 0.7 BD. Recordings from electrodes in the adductor muscle of the pectoral fin (Add.) and along the left side of the body (1–5) are shown in full colour. Recordings from electrodes along the right side of the body are shown directly below in lighter shades. 1–5 are positions spaced equidistantly in red axial muscle from the posterior edge of the pectoral fin lobe to the anterior edge of the anal fin. See Materials and Methods for precise location. All traces are from a single individual.

While many studies have investigated how fishes move in a terrestrial environment (reviewed in Pace and Gibb, 2014; Lutek et al., 2022), less is known about how motor control and sensory information elicit terrestrial behaviour in amphibious animals (Cabelguen et al., 2010; Foster et al., 2018; Gillis, 2000; Horner and Jayne, 2014; Perlman and Ashley-Ross, 2016). Studies that investigate fish muscle activation in fully aquatic or terrestrial environments often report statistically significant differences in muscle activity parameters between the two environments (Foster et al., 2018; Gillis, 2000; Horner and Jayne, 2014; Perlman and Ashley-Ross, 2016). In general, terrestrial axial muscle activity has a higher intensity and longer duration, and the adductor of the pectoral fin may change from a single burst pattern during swimming to a double burst pattern during walking (Foster et al., 2018; Gillis, 2000; Horner and Jayne, 2014; Perlman and Ashley-Ross, 2016). Thus, terrestrial locomotion in amphibious fishes appears to require more muscle effort, likely to overcome the increase in friction between the body and substrate that accompanies the transition to land (Fig. 1A,C). While these studies speak to the discrete difference between fully aquatic and fully terrestrial biomechanics, they do not address the possibility of a behavioural continuum across the ecologically relevant transition between water and land. Subtle changes in water level will alter environmental physical forces and resultant sensory feedback, which help shape motor control and resultant behaviour (Fig. 1A–C).

Few studies have investigated the kinematics of the aquatic–terrestrial transition in amphibious animals. Salamanders moving up or down a slope switch between walking and swimming while partially submerged, but precisely when this transition occurs varies between trials (Ashley-Ross and Bechtel, 2004). Elongate *Erpetoichthys calabaricus* (ropefish), when in intermediate water depths, move using behaviours that combine the characteristics of aquatic and terrestrial locomotion (Pace and Gibb, 2011). The more classically ‘fusiform’ *K. marmoratus* (mangrove rivulus) use three separate behaviours (launches, squiggles and pounces) to move between aquatic and terrestrial environments, depending on the physical environment and desired outcome (Pronko et al., 2013). Each species navigates the aquatic–terrestrial transition in a unique way, possibly as a result of differences in which propulsive elements are used for locomotion. For example, salamanders swim using axial undulations and walk using their legs (Cabelguen et al., 2003) whereas ropefish use primarily axial undulations to move in both aquatic and terrestrial environments (Pace and Gibb, 2011). Determining how, and in which environments, body and fin motion and muscle activity change sheds light on the complex interactions of axial/appendicular motor control and the physical environment in amphibious animals.

*Polypterus senegalus* is the extant species closest to the common ancestor of actinopterygians and sarcopterygians and is capable of terrestrial locomotion powered by coordinated pectoral fin and body movements. Previous work demonstrates marked kinematic and muscle activity differences between swimming and walking in this species (Foster et al., 2018; Standen et al., 2014, 2016). During swimming, pectoral fins are moved in-phase with little evidence of adductor/abductor co-contraction (Foster et al., 2018; Standen et al., 2014, 2016); body undulation is minimal with little or no red muscle activity in the anterior half of the body. When moving over land (i.e. walking), pectoral fins move out-of-phase with a large range of motion (RoM) and the body exhibits large lateral undulations (Standen et al., 2014, 2016). These movements are powered by activation of the adductor muscle in the pectoral fin that suggests co-contraction with the abductor, likely leading to fin stiffening, and increased red muscle recruitment at the middle of the body (Foster et al., 2018). However, these investigations looked at behaviour only at the extremes of the aquatic–terrestrial environmental continuum. Thus, they also do not address the possibility of a gradual behavioural or control transition between swimming and walking that varies with an environmental gradient.

To further investigate the swimming-to-walking transition, we exposed *P. senegalus* to stepwise water depths that span the aquatic–terrestrial transition. To capture a finer resolution across those environments where changes in physical forces within the environment are largest, we paid particular attention to the depths at which buoyancy likely gives way to gravity as the dominant force (Fig. 1B). In this way, we tested the hypothesis that the transition from swimming to walking in *P. senegalus* is actually a continuum (the continuum hypothesis) and predict that, as water depth decreases, there will be a gradual shift in kinematics and motor control patterns. The continuum hypothesis exists in contrast to the alternative hypothesis (the discrete hypothesis), which, based on the existing literature (Standen et al., 2014; Foster et al., 2018), posits that there is a discontinuity between walking and swimming and predicts that a discrete switch exists in both kinematics and muscle activation pattern at a critical water depth.

## MATERIALS AND METHODS

### Animals

The results presented here are from two experiments on two different groups of *Polypterus senegalus* Cuvier 1829. In one group of fish (kinematics group; *n*=5) we recorded kinematics across a gradient of eight water depth treatments, spanning fully aquatic to fully terrestrial, measured as a proportion of body depth (BD): 3.0, 2.0, 1.1, 1.0, 0.9, 0.7 (height of the top of the eyes), 0.1 (height of the mouth) and 0.0 BD (no water above the wetted substrate) over the course of several days (Movies 1–3). BD was standardized as the distance from the ground to the tallest part of the dorsal surface (not including the dorsal fin) of each fish. The kinematics group had a mean (±s.e.m.) total body length (BL) of 95.5±3.20 mm and a mean mass of 5.01±0.54 g. Each fish in the kinematics group was filmed in no more than three conditions each day and on no more than 3 days each week to avoid fatigue effects. In a second group of fish [electromyography (EMG) group; *n*=4, BL 123.5±3.66 mm, mass 9.29±0.89 g], we recorded kinematics and muscle activity in depths of 3.0, 1.1, 1.0, 0.9 and 0.7 BD (Fig. 1). We were limited to these depths as EMG experiments in *P. senegalus* must be completed in a single day. Each fish in the EMG group was recorded in order from greatest to least BD and permitted time to rest between conditions and bouts of filming. Unless otherwise stated, methods are applicable for both groups of animals and all values are reported as means±s.e.m.

*Polypterus senegalus* were acquired from the pet trade (AQUAlity Tropical Fish Wholesale Inc., Mississauga, ON, Canada) and housed in individual recirculating (10% water change each week) tanks on a 12 h:12 h light:dark cycle at 25–26°C. Except for one fish from the kinematics group in 0.1 BD (which completed three cycles), all fish completed at least five tailbeat cycles at each water depth. Experiments were performed according to the University of Ottawa Animal Care Protocol BL-1926.

### High-speed videography

For the kinematics group, trials were filmed on a 21×10 cm Plexiglas board covered tightly in cheesecloth to increase traction and placed in a 38 l tank. High-speed video (500 frames s^−1^) was captured from a single side view (Fastec IL5 high-speed camera, Fastec Imaging, San Diego, CA, USA) and two top views, using two Photron Fastcam Mini UX high-speed cameras (Photron USA Inc., San Diego, CA, USA) suspended above the platform and angled approximately 45 deg from each other. These three camera views were calibrated for three-dimensional (3D) analysis using a 42-point calibration object in DLTcal5 (Hedrick, 2008) in MATLAB (version R2018/2019/2020, The MathWorks Inc., Natick, MA, USA).

For the EMG group, trials were filmed on a 35×35 cm Plexiglas board covered tightly in cheesecloth and placed in a 34 l tank. High-speed video (500 frames s^−1^) was captured from the top view (Photron Fastcam Mini UX high-speed camera) and three side views (one Photron Fastcam Mini UX high-speed camera and two Fastec IL5 high-speed cameras). These four camera views were calibrated for 3D analysis using a 44-point calibration object in DLTcal5 (Hedrick, 2008).

In all videos, the nose, tail tip and middle of the distal edge of the lobe of the right pectoral fin were digitized manually in DLTdv6 (Hedrick, 2008) to get 3D coordinates. For the kinematics group, the tip of the left pectoral fin lobe was also digitized as above to calculate fin RoM. For both datasets, top views were binarized and fish midlines were tracked automatically using custom-written MATLAB code. One-hundred evenly spaced points along the fish's body were selected from these midlines and used to reconstruct the fish midline coordinates in the *x*–*y* plane. Timing of the beginning of fin adduction and abduction was identified manually in the open-source image analysis platform Fiji (Schindelin et al., 2012).

### Kinematics analysis

To facilitate comparisons across water depths, stroke timing was calculated in two ways: (1) as pectoral fin stroke, defined by right pectoral fin motion (start of adduction=stroke start, start of abduction=mid-stroke), and (2) tail stroke, defined by tail motion (start of tail swing to the right=stroke start, start of tail swing to the left=mid-stroke). As swimming *P. senegalus* show no coordination between body and pectoral fins in both previous and present datasets (Foster et al., 2018; Standen et al., 2014), left fin timing was defined relative to the pectoral fin stroke and timing of maximum left and right amplitude for the body was defined relative to the tail stroke.

For each trial, we calculated the following variables: locomotion speed (BL s^−1^), curvature coefficient (a measure of overall fish curvature; BL), swing distance (total distance a body point travels in the *x*–*y* plane from maximum left amplitude to maximum right amplitude; BL), wave frequency (cycles s^−1^), pectoral fin frequency (cycles s^−1^), pectoral fin RoM (maximum fin angle−minimum fin angle; deg) and nose elevation (BL). Magnitude variables were standardized to BL to permit comparisons between individuals. Curvature coefficient (modified from Brainerd and Patek, 1998) was calculated as follows:
(1)

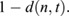


In Eqn 1, *d*(*n*,*t*) is the minimum linear distance between nose and tail during a locomotor cycle standardized to BL. Thus, a curvature coefficient of 1 represents the nose and tail touching during locomotion and a value of 0 represents a perfectly straight fish. Pectoral fin frequency was calculated based on the pectoral fin stroke as defined above. Pectoral fin angle was defined as the angle between the nose, back of skull and tip of fin lobe (as in Foster et al., 2018, and Lutek and Standen, 2021). Both fin RoM and nose elevation showed no differences in magnitude between the left and right side; thus, a mean value for each trial calculated from both left- and right-side data was used in the final analysis. All linear variables use trial averages as individual observations. We report the start time of left fin adduction, and the timing of maximum amplitude of the body to the left and to the right in polar coordinates. Maximum body amplitude timing was assessed only at 40%, 60% and 80% BL as body amplitude is minimal at more anterior positions in higher water depths. As polar coordinates are relative and circular, they facilitate focus on the phase differences between strokes. All variables were calculated using custom-written MATLAB code. Because the kinematics of the two experimental groups of fish were similar, we report only the kinematics group data for all kinematic variables, as this group covers the largest number of water depths.

### EMG

For the EMG group, each fish was anaesthetized prior to surgery using 200 mg l^−1^ buffered tricaine methanesulfonate (MS222, Syndel Laboratories Ltd, Nanaimo, BC, Canada). Throughout surgery, fish were kept moist and sedated using fish housing water or anaesthetic as required. Two-pronged fine wire hook electrodes were fashioned from 0.051 mm bi-filament wire (California Fine Wire Company, Grover Beach, CA, USA) and implanted percutaneously using 27-gauge needles (Sigma-Aldrich, St Louis, MO, USA). Electrodes were implanted in the middle of the pectoral fin adductor, in five locations spaced evenly along the left-side red body muscle between the pectoral fin lobe and anal fin (19.8±0.73% BL, 33.1±0.51% BL, 42.8±0.69% BL, 57.4±0.39% BL and 67.4±0.74% BL), and opposite positions 2 and 4 on the right side of the body (as in Lutek and Standen, 2021). Following the placement of all electrodes, the wires were sutured to the dorsal spines to reduce strain on the electrodes. Individuals were allowed to recover in fish housing water prior to performing locomotor trials. We recorded EMG signals using a Grass P511 AC Amplifier (Natus Neurology, San Carlos, CA, USA) passed through a Powerlab 16/35 digital-to-analog converter (ADInstruments, Colorado Springs, CO, USA). Signals were recorded at 10 kHz with a 60 Hz notch filter (to eliminate ambient electrical noise) in Lab Chart 8 (ADInstruments). Signals were then filtered with a 40–4000 Hz bandpass filter and analysed using custom-written MATLAB code. Video and EMG recordings were synchronized using an external trigger. Following recordings in all depths, each fish was euthanized with an overdose of buffered MS222 (417 mg l^−1^ in fish housing water). Then, mass and BL were recorded and electrode position was confirmed by dissection.

### EMG analysis

Muscle burst start and stop times were identified from EMG traces manually. Note that two fish in 0.7 BD did not have consistent bursts of muscle activity and so onsets and offsets were not identified for these individuals (Fig. S1). Using the onset and offset timings, we calculated burst duration as a percentage of the tailbeat cycle (EMG duty factor) and rectified integrated area (EMG RIA; sum of rectified muscle signals within a burst of activity reported as a percentage of the theoretical maximum RIA to permit comparison between individuals). This theoretical maximum was calculated as the mean of the top 5% of muscle signals for a given electrode within each fish multiplied by the duration of an individual burst of muscle activity. EMG duty factor and EMG RIA showed no difference between left-side and right-side electrode positions and so are reported only for the left-side electrodes. The timing of body EMG onsets and offsets for electrodes on the left side of the body at positions 3, 4 and 5 is reported relative to the tailbeat cycle in polar coordinates. We looked only at these sites to match the body sites assessed for consistent maximum kinematic amplitude timing. Contralateral co-activation of left- and right-side muscles was quantified by comparing the timing of muscle bursts at the same position on opposite sides of the body (electrode sites 2 and 4).

### Statistical analysis

All linear variables were analysed using linear mixed effects models created in R using the nlme package (https://CRAN.R-project.org/package=nlme; http://www.R-project.org/). Models for each linear variable included fixed effects for swim speed, water depth and site (as dictated by model comparisons) and fish identity as a random effect (full model fixed effects: dependent variable∼speed×depth×site, random effects: ∼1 | fish) (see Table S1 for specific models for each dependent variable). Residuals were checked for normality (visual inspection of a histogram) and equal variance (Levene's test) across water depths. When necessary, models were adjusted to accommodate unequal variance across water depth or site using the varIdent function (https://CRAN.R-project.org/package=nlme; Table S1). ANOVA-type output tables (Type III Sums of Squares) were generated using the anova.lme function (https://CRAN.R-project.org/package=nlme). *R*^2^ values were estimated using the MuMIn package (https://CRAN.R-project.org/package=MuMIn). Two-tailed pairwise multiple comparisons were performed as appropriate based on the estimated marginal means (and associated s.e.m.) from each model. These pairwise comparisons were Bonferroni corrected based on the number of comparisons performed within each kinematic variable to account for type I error.

Cycle timing (polar coordinates) was analysed using standard polar statistics (similar to Standen, 2008; Standen et al., 2014) using custom-written MATLAB code based on Zar (1999) and Batschelet (1981). First, data were tested for a non-uniform distribution using the Hermans–Rasson (HR) test (Landler et al., 2019). This test is sensitive to any deviation from a uniform distribution, including non-axial, bimodal distributions (as observed in 0.7 and 0.1 BD for left pectoral fin adduction timing in our dataset). If a variable was non-uniform (HR *P*<0.05), it was then tested for a von Mises distribution. The von Mises distribution is the circular distribution analogue of the linear normal distribution and is a requirement for running Rayleigh's test for a unimodal distribution. Thus, if the data had a von Mises distribution, data were tested for a unimodal distribution using Rayleigh's test. The combination of the HR and Rayleigh's test in this order distinguishes between non-uniform, multimodal distributions [HR *P*<0.05, Rayleigh's test (R) *P*>0.05] and non-uniform, unimodal distributions (HR *P*<0.05, R *P*<0.05). A non-uniform, multimodal distribution suggests that data timing occurs consistently at multiple time points in a kinematic cycle (e.g. an event that occurs twice with regular timing during a kinematic cycle). A non-uniform, unimodal distribution suggests that data timing occurs consistently at a single time point in the kinematic cycle. If the data had a non-uniform, unimodal distribution, an angular mean and angular variance were calculated. Neither angular mean nor angular variance was calculated for multimodal distributions.

We additionally investigated whether left pectoral fin adduction timing was solely in-sync or out-of-sync within each trial by testing for a von Mises distribution, a non-uniform unimodal distribution (Rayleigh's test) and calculating a mean and angular variance when the Rayleigh's test gave *P*<0.05. As we were looking only for unimodal in-sync or out-of-sync behaviour in each trial, the HR test is unnecessary here. For nose elevation, we tested for differences in angular dispersion (the absolute difference between each observation and the circular mean in each depth) between water depths using a Kruskal–Wallis test to determine whether kinematic timing became more or less variable across water depths (Zar, 1999). For the timing of maximum body amplitude and the timing of body muscle onset and offset, we tested for differences in angular mean between water depths using Watson–Williams tests (Zar, 1999). Both Kruskal–Wallis and Watson–Williams tests were followed by pairwise comparisons as needed. These pairwise comparisons were Bonferroni corrected (as for linear statistics above) to account for type I error.

## RESULTS

### Kinematic changes across water depths

**Fig. 2.**
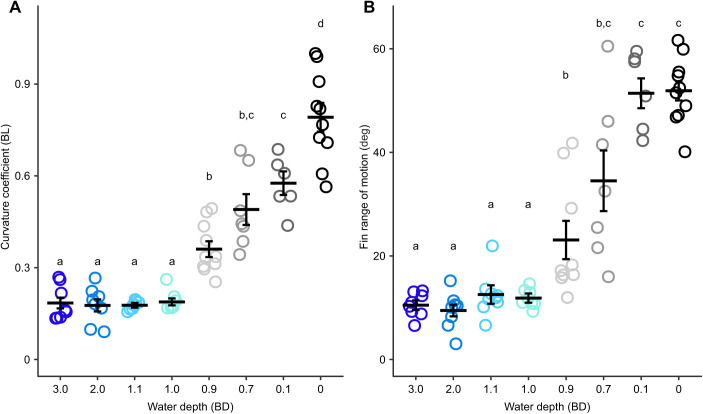
**Fin and body movement changes across water depths.** (A) Curvature coefficient (BL, body length) and (B) fin range of motion show a significant increase when water depth drops to 0.9 BD (*N*=5 individuals). Different lowercase letters denote means that are significantly different between water depths. Individual points are the mean for each trial; thick horizontal lines and error bars are the estimated marginal mean±s.e.m for each water depth.

**Fig. 3.**
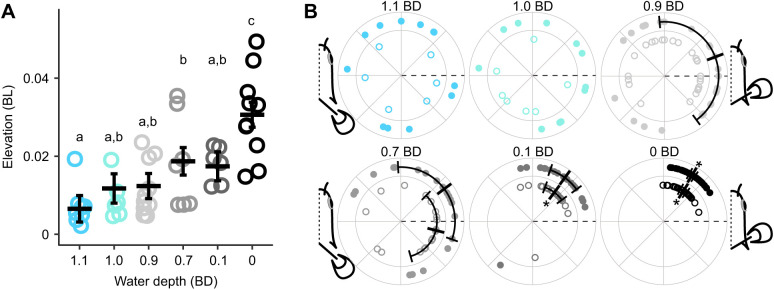
**Nose elevation increases and is less variable below 0.1 BD.** (A) Nose elevation magnitude and (B) timing relative to the pectoral fin stroke (*N*=5 individuals). Each point in A is a trial mean. Estimated marginal means and their s.e.m. are shown by the thick horizontal bar and error bars. Different lowercase letters denote means that are significantly different between water depths. In B, the timing of maximum elevation during swing to the right (filled circles) and left (open circles) is plotted relative to right and left fin cycles, respectively. Nose elevation timing is directional for elevations to the right in 0.9 BD and for both left and right elevation in 0.7, 0.1 and 0.0 BD. When nose elevation timing is directional, a circular mean±angular variance is plotted (thick bar and error bars). Nose elevation timing is less variable in 0.1 and 0.0 BD than in 0.9 BD and higher. Each point is an individual maximum elevation. 0 deg (dashed line) is the start of fin adduction for the right and left fins; 180 deg is the start of fin abduction for the right and left fins. Asterisks denote significantly lower angular dispersion than at water depths above 0.1 BD.

We found discrete changes in kinematics across water depths. Mean locomotion speed was largely consistent across water depths; however, there was a slight increase at 0.9 BD and a slight decrease at 0.1 and 0.0 BD relative to other depths (Tables 1 and 2). To capture this variation in speed, it was left in select models below. Curvature coefficient increased as water depth decreased below 1.0 BD (Tables 1 and 2, Fig. 2A). Swing distance increased significantly with speed and towards the posterior of the fish (Tables 1 and 3). Swing distance tended to increase as water depth decreased (Tables 1 and 2). Wave frequency increased significantly as fish moved faster (Table 1). There was no difference in wave frequency across sites (Tables 1 and 3). Across water depths, wave frequency was significantly higher at 1.1, 0.7 and 0.1 BD than at 3.0 BD (Tables 1 and 2). The effect of locomotion speed on pectoral fin frequency depended on depth (Table 1). Pectoral fin frequency increased slightly as water depth decreased from 3.0 to 0.9 BD, then decreased as water depth dropped to 0.0 BD (Tables 1 and 2). Pectoral fin range of motion increased significantly as water depth decreased below 1.0 BD and increased significantly again as water depth decreased below 0.1 BD (Tables 1 and 2, Fig. 2B). Nose elevation became significantly greater in 0.1 and 0.0 BD (Tables 1 and 2, Fig. 3A).

**Table 1.**
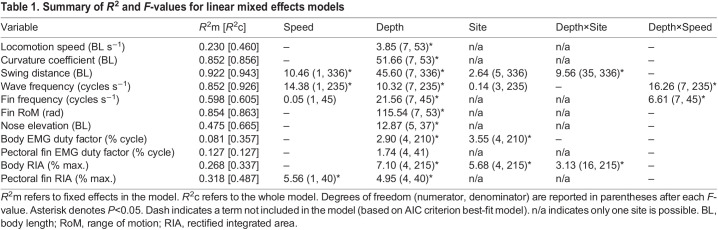
Summary of *R*^2^ and *F*-values for linear mixed effects models

**Table 2.**
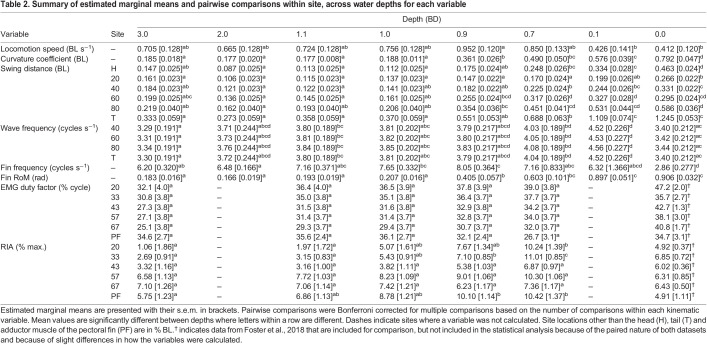
Summary of estimated marginal means and pairwise comparisons within site, across water depths for each variable

**Table 3.**
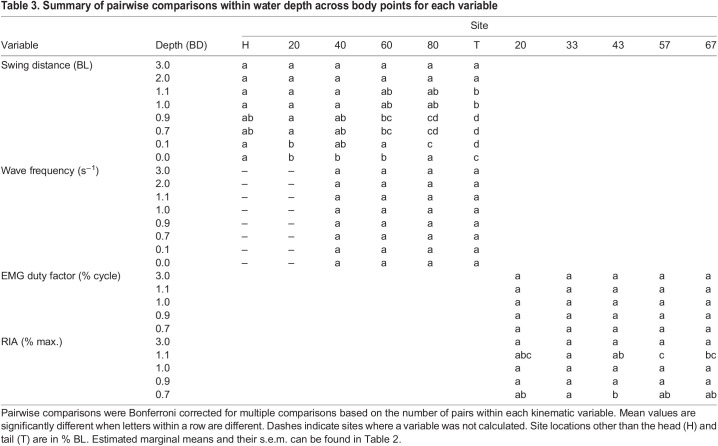
Summary of pairwise comparisons within water depth across body points for each variable

**Fig. 4.**
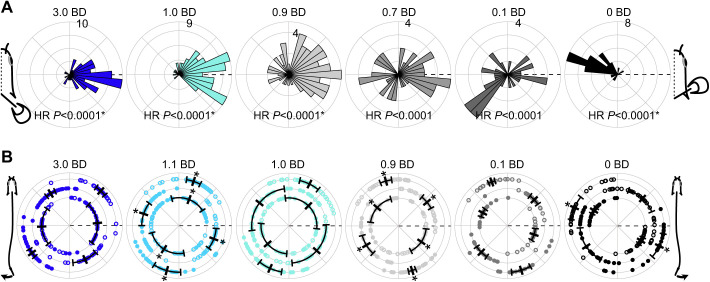
**Fin and body timing changes across water depths.** (A) Left pectoral fin timing; 0 deg is the start of right fin adduction (dashed line); 180 deg is the start of right fin abduction (*N*=5 individuals). The timing of the start of left pectoral fin adduction is binned in 10 deg increments and presented as histograms. The Hermans–Rasson (HR) *P*-value is shown at the bottom of each plot. Asterisks denote cases where the Rayleigh's test *P*-value is <0.05, indicating data have a unimodal distribution. (B) Left (filled circles) and right (open circles) maximum body amplitude relative to the tail stroke; 0 deg is the start of tail swing to the right (dashed line); 180 deg is the start of tail swing to the left. Vertical bars and error bars represent the circular mean and angular variance. Data are shown for 40%, 60% and 80% BL (inner to outer ring in each plot). Asterisks denote angular means that are statistically different from the same position in the next greatest water depth shown.

In general, the timing of the left pectoral fin relative to the right pectoral fin was either in-phase or out-of-phase, with few strokes having an intermediate timing (Fig. 4A). Pectoral fin timing was non-uniform (HR *P*<0.0001) and unimodal (R *P*<0.05) in all conditions except 0.7 and 0.1 BD (R *P*>0.05; Fig. 4A) where trials had consistent timing (Fig. S2) but were both in-phase and out-of-phase within a given trial in these conditions, sometimes within an individual fish. For trials that were too short or had variable timing, and thus had a Rayleigh's test *P*-value >0.05, individual data are still shown, but no mean or confidence interval could be calculated for these data (Fig. S2). In 3.0 and 2.0 BD, the timing of leftward maximum body amplitude was consistent, coincident with maximum leftward tail amplitude for 40% BL, just before maximum rightward tail amplitude for 60% BL and just after maximum rightward tail amplitude for 80% BL (Table 4, Fig. 4B), and likewise for the timing of rightward maximum body amplitude relative to maximum leftward tail amplitude. The timing of maximum leftward and rightward amplitude at all positions shifted significantly later in the cycle at 1.1 BD, compared with 3.0 and 2.0 BD, and maintained this timing in 1.0 BD (Table 4, Fig. 4B). Maximum leftward and rightward amplitude at all positions shifted later again in 0.9 BD, relative to 1.1 and 1.0 BD, and remained consistent through to 0.1 BD (Table 4, Fig. 4B). In 0.0 BD, the timing of maximum leftward and rightward amplitude at 40% and 60% BL was consistent with timing in 0.9–0.1 BD, but the timing of 80% BL shifted later in the cycle again, relative to 0.9–0.1 BD. The timing of maximum nose elevation, relative to the pectoral fin stroke cycle, was directional (just after the beginning of the stroke) in 0.9 BD (nose elevation to the right only), 0.7, 0.1 and 0.0 BD (Fig. 3B). The angular dispersion of nose elevation decreased as water depth decreased for nose elevation to the right (χ^2^=41.45, d.f.=5, *P*<0.0001) and to the left (χ^2^=50.42, d.f.=5, *P*<0.0001) (Fig. 3B). Dispersion was significantly lower in 0.1 BD (nose elevation to the left) and 0.0 BD (nose elevation to the right and left) than in 0.9 BD and above (Fig. 3B).

**Table 4.**
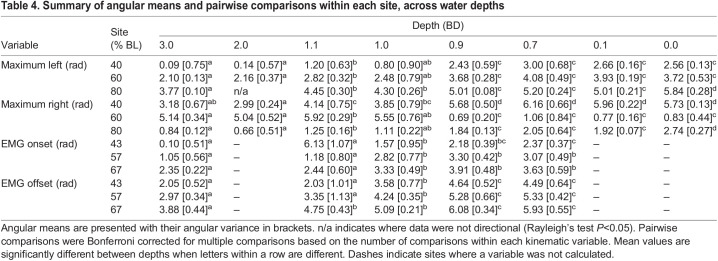
Summary of angular means and pairwise comparisons within each site, across water depths

### Muscle activity changes across depths

**Fig. 5.**
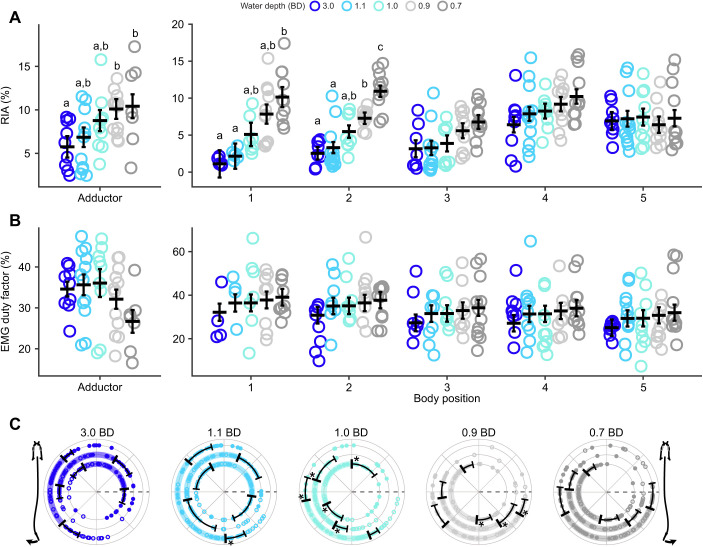
**Muscle activity changes across water depth in *P. senegalus*.** (A) Rectified integrated area (RIA) increases as water depth decreases for the fin adductor muscle and the anterior two positions on the body. (B) EMG duty factor shows no statistically significant changes as water depth decreases. (C) Muscle onset (filled circles) and offset (open circles) for axial red muscle. In A and B, small points are trial means and the estimated marginal mean±s.e.m. is presented as a thick horizontal line and error bars. Data are shown for the fin adductor muscle and body positions 1–5, as defined in Materials and Methods. Different lowercase letters denote means that are significantly different between water depths within a position. In C, small points are individual observations; vertical bars and error bars represent the circular mean and angular variance (angular variance only shown on one side of the mean). The portion of the cycle when each muscle is active is represented by a bar of colour between the mean onset and offset time. Data are shown for body positions 3, 4 and 5 (inner to outer ring in each plot; see Materials and Methods for details). *N*=4 individuals for all depths except 0.7 BD, where *N*=2; see Materials and Methods for details. Asterisks denote angular means that are statistically different from the angular mean at the same position in the next greatest water depth shown.

EMG RIA at body positions 1 and 2 and in the adductor muscle of the pectoral fin increased significantly as water depth decreased but showed no significant changes at body positions 3–5 (Tables 1, 2 and 3, Fig. 5A). EMG duty factor remained constant across all fin and body electrode positions and in all treatments (Tables 1, 2 and 3, Fig. 5B). Note, however, that two individuals had muscle activity in the adductor of the pectoral fin that was distinctly longer, with an intensity that varied across the active duration of the stroke (reminiscent of walking traces reported previously; Foster et al., 2018), and showed inconsistent and indistinct muscle activity in 0.7 BD (Fig. S1). The timing of red axial muscle onset along the left side of the body at sites 3, 4 and 5 generally shifted later in the tailbeat cycle as depth decreased (Table 4, Fig. 5C). Right- and left-side body muscle was rarely active at the same time. At position 2, right and left sides were active simultaneously for the following number of bursts in each condition, 3.0 BD – 0/35, 1.1 BD – 2/49, 1.0 BD – 4/38, 0.9 BD – 5/37 and 0.7 BD – 2/41; and for position 4, 3.0 BD – 0/45, 1.1 BD – 1/52, 1.0 BD – 0/44, 0.9 BD – 2/37 and 0.7 BD – 2/42.

## DISCUSSION

### Aquatic and terrestrial locomotion in elongate fishes

*Polypterus senegalus* exhibited discrete kinematic changes at water depths where the body and pectoral fin were first obligated to interact with the substrate. Despite this, axial red muscle always carried a travelling wave of muscle activation from anterior to posterior with little contralateral muscle activity overlap. Additionally, muscle effort gradually increased as water depth decreased. As red axial muscle always carried an anterior-to-posterior wave of muscle activity and increases in muscle effort were continuous, we suggest that walking in *P. senegalus* is accomplished by swimming-like body muscle activity. The different kinematic output between swimming and walking can be explained because, although the muscle activity pattern is similar, interactions between the fish and the external environment constrain axial red muscle contractions, resulting in modified kinematic performance. The increased friction and lack of buoyancy in transitional environments may change the range of muscle lengths over which axial muscles are active and/or alter the terrestrial waveform by physically hindering kinematic movement. Thus, changes in axial muscle activity across the aquatic–terrestrial transition support the continuum hypothesis despite discrete kinematic changes.

In contrast, pectoral fins show a starker contrast in behaviour between swimming and walking modes. As fish move into shallow water (0.7 and 0.1 BD), their left and right pectoral fins switch discretely from in-phase to out-of-phase movements and the adductor muscle of the pectoral fin undergoes a much longer activation duration (in seconds) because pectoral fin beat frequency is lower during walking than during swimming (Tables 1 and 2). Although this appears to support the discrete hypothesis, that fish are changing their motor control pattern to accommodate novel walking behaviour, it does not necessarily suggest that a different neural circuit is used for fin motion during walking. Here, increases in pectoral fin muscle activation duration are not accompanied by a change in EMG duty factor, meaning the timing of individual pectoral fin patterns within the step cycle is similar between most walking steps (see ‘Mechanisms of muscle activity modulation’, below, for details) and swimming strokes. In addition, asymmetric pectoral fin coordination is common during unsteady (e.g. turning, acceleration, backwards swimming) aquatic behaviours in both *P. senegalus* (K.L. and E.M.S., unpublished observations) and other fish species (e.g. Drucker and Lauder, 2002; Drucker and Lauder, 2003; Gerstner, 1999), suggesting this is not a novel terrestrial control pattern but rather a co-opting of existing aquatic manoeuvring patterns. Further, a discrete switch between limb use and limb adduction against the body is seen in both live and modelled salamander locomotion and may be due to a single control circuit (Cabelguen et al., 2003; Ijspeert et al., 2007). A single neural circuit may elicit both walking and swimming in salamanders if limbs are tonically adducted above a threshold descending neural drive (Ijspeert et al., 2007). Thus, although there is a large and apparently discreet change in the left–right coordination of pectoral fin kinematics at particular water depths, this may be driven by a gradual increase in descending drive, and a particular neural threshold that switches pectoral fin movements from in-phase to out-of-phase. The gradual increase in drive could, for example, be driven by sensory feedback based on changes in the relative magnitude of gravitational and buoyant forces across the environmental continuum. If an interneuron changes its firing pattern (or starts firing) above a given feedback threshold, this could elicit a change in coordination of the pectoral fins. If pectoral fin kinematic changes are driven by a single neural circuit with the necessary neural thresholds, as we suggest and as observed in salamanders, then despite a discrete change in outward kinematics, these results would be consistent with the continuum hypothesis.

How does this view of walking compare to terrestrial muscle activity for other elongate fishes? Comparisons of muscle activity between aquatic and terrestrial locomotion in *P. senegalus*, in eels and in lungfish show similar changes in muscle activity (Foster et al., 2018; Gillis, 2000; Horner and Jayne, 2014). All three species tend to have higher anterior body RIA, longer body EMG duration and body EMG onset later in the kinematic cycle during terrestrial locomotion. Our study shows similar muscle activity changes, but in a gradual manner as water depth decreases. This raises the possibility that these three species accomplish terrestrial locomotion using a swimming-like axial muscle activity pattern that is highly influenced by interaction with the substrate.

While this is the first report of fish muscle activity in environments that span the water–land interface, previous kinematic work in the elongate ropefish provides an interesting point of comparison for our results. Both aquatic and terrestrial locomotion in ropefish are primarily powered by body movements. In contrast, *P. senegalus* power swimming primarily with pectoral fin movements (and body undulation gradually increases as swimming speed increases) whereas both pectoral fins and the body power terrestrial locomotion. Despite this difference, both species appear to show an increase in body curvature with decreasing water depth (Pace and Gibb, 2011). Although EMG is not recorded for ropefish transitioning through different water depths, the ropefish kinematic response is like that of *P. senegalus* and suggests that ropefish may also use a swimming-like axial muscle activity pattern during terrestrial locomotion. Ultimately, investigating muscle activity across the aquatic–terrestrial transition in multiple species, including water depths that bridge the gap between 0.7 and 0.0 BD in *P. senegalus*, is essential to test the continuum hypothesis across these amphibious fishes.

### Mechanisms of muscle activity modulation

**Fig. 6.**
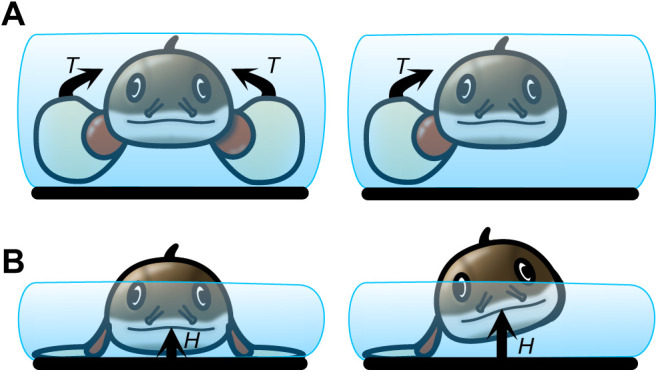
**Environmental forces influence locomotor mode in *P. senegalus*.** Schematic diagrams of the influence of in- and out-of-phase pectoral fin movements in (A) an aquatic environment and (B) 0.7 BD. In-phase pectoral fin movements (left) result in balanced torque and thrust forces on the fish in an aquatic environment (A), but cannot lift the head off the ground in 0.7 BD (B). Out-of-phase pectoral fin movements (right) result in unbalanced torque and thrust forces on the fish in an aquatic environment (A), but allow the body to roll and lift the nose of the fish higher off the ground in 0.7 BD (B). *T*, torque; *H*, height.

That a fish can have a discreet change in behaviour with a continuous change in muscle activation emphasizes the role of environmental constraint on the resulting kinematics. Compared with swimming, body bending and fin range of motion increased significantly and peak body bending occurred later in the tailbeat cycle when water dropped to 0.9 BD. This coincides with the environment where the weight of the fish is no longer fully supported by buoyant forces (Fig. 1B). This increase in body and fin motion is likely required to increase locomotor force and overcome higher resistance resulting from the slow increase in frictional loading of the belly on the ground. As water depth decreased further, to a point where the pectoral fin must interact with the substrate (0.7 BD), fin kinematic timing changed from in-phase to out-of-phase oscillations. The switch to out-of-phase pectoral fin movements allowed fins to coordinate with the increase in body curvature, helping to lift the anterior body off the ground (Figs 2 and 3). This likely created higher ground reaction forces to facilitate lift and forward movement. Presumably, there is a threshold water depth below which thrust production using in-phase fin movements can no longer overcome the increased ground friction. If *P. senegalus* provide propulsion for steady locomotion using either in- or out-of-phase fin movements, the discrete use of these behaviours in different environments could facilitate the most effective locomotion based on the physical limitations of each environment (Fig. 6). For example, torque and thrust generated by in-phase pectoral fin movements in an aquatic environment would be balanced in comparison with forces generated by fins moving out-of-phase (Fig. 6A). Unbalanced forces would likely decrease swimming efficiency. In contrast, in-phase pectoral fin movements in water depths <0.7 BD cannot lift the body of *P. senegalus* off the ground, thus rendering them ineffective for terrestrial locomotion (Fig. 6B). Out-of-phase fin planting movements in a terrestrial environment combined with forces generated by the body allow the fish to roll, lift the head off the ground, and reduce friction between the body and substrate (Fig. 6B). Partitioning locomotor modes based on the ability to produce forward movement through a particular environment may be a common characteristic of transitional movements in amphibious fishes. *Kryptolebias marmoratus* also appear to select particular transitional behaviours based on the environment they are in (Pronko et al., 2013) and ropefish show individual bouts of locomotion that are more swimming- or walking-like when 75% emersed, potentially as a consequence of microfluctuations in the testing environment (Pace and Gibb, 2011). A discrete separation of locomotor strategies in these transitional environments is likely the result of a set of complex (and possibly conflicting) physical constraints and sensory cues, and such behavioural switches may be easily accomplished using neural thresholds (in line with the continuum hypothesis) as described above.

Environmental constraint combined with subtle antagonistic muscle activation may also explain how two different muscle activity patterns in the adductor of the pectoral fin at 0.7 BD (Fig. S1) result in a single kinematic function. One pattern exhibits a more continuous activation of the adductor muscle while the other shows clear independent bursts, but both patterns are associated with a clear step cycle for the fin. For a continuous muscle contraction to be active during both adduction and abduction in a fin cycle, the muscle must operate across both lengthening and shortening regimes. This means that the muscle is working against the constraint of the environment, lengthening under the weight of the body against the ground, or working against the contraction of antagonistic muscles of the fin. The occurrence of two muscle activation patterns that result in the same kinematic output suggests that fish can employ multiple ‘strategies’ to achieve the same kinematic function. Thus, effective locomotion here is the result of an interaction between the existing musculoskeletal anatomy of the animal and the physical constraints of the environment itself, which confines outward behaviour to a single successful kinematic function. It is interesting to speculate that because the extended duration of muscle activity in the adductor of the pectoral fin that we observed occasionally in 0.7 BD is reminiscent of ‘double’ bursts reported previously during walking (Foster et al., 2018), at water depths below 0.7 BD, the adductor muscle of the pectoral fin may always behave in this extended duration capacity.

### Neuromuscular control in novel environments

A swimming-like axial motor pattern appears to be sufficient to produce walking in *P. senegalus*, which suggests the utility (and possible evolutionary advantage) of a single pattern that can be used in several environments. *Polypterus senegalus* also use modified swimming axial muscle activity in high viscosity (Lutek and Standen, 2021). However, in such high-resistance aquatic environments, they increase posterior body muscle effort (rather than anterior body muscle effort as water depth decreases), suggesting different modifications of the base swimming pattern in response to novel aquatic and terrestrial environments. If the same motor control circuit that coordinates swimming can be used in more resistive environments, regardless of the medium, then exploring novel environments becomes easier, even when there is no prior exposure to that environment (as seems likely the case for land in *P. senegalus*; Du et al., 2016)*.* That the body and pectoral fins change kinematic timing in different depths (at 0.9 BD and 0.7 BD, respectively) may also suggest a weak neural coupling between the two. Subtle timing changes may afford these fish additional flexibility in their behavioural repertoire by allowing independent modulation of the body and pectoral fins. Overall, our results suggest that in *P. senegalus*, axial and pectoral fin muscle activity across the aquatic–terrestrial transition is consistent with the continuum hypothesis and that discrete changes in outward kinematics are the result of physical environmental constraint acting on propulsive structures. Further investigations of amphibious fishes in environmental gradients, such as we have done here, will help determine whether this principle holds across a variety of fishes.

## Supplementary Material

10.1242/jexbio.243902_sup1 Supplementary information
