# Supplementary material for: Behaviour and muscle activity across the aquatic–terrestrial transition in Polypterus senegalus
Source: J Exp Biol. 2022 Dec 9;225(23):jeb243902. doi: 10.1242/jeb.243902 (PMC9789406; doi:10.1242/jeb.243902)
Supplement: Supplementary information [file jexbio-225-243902-s1.pdf]

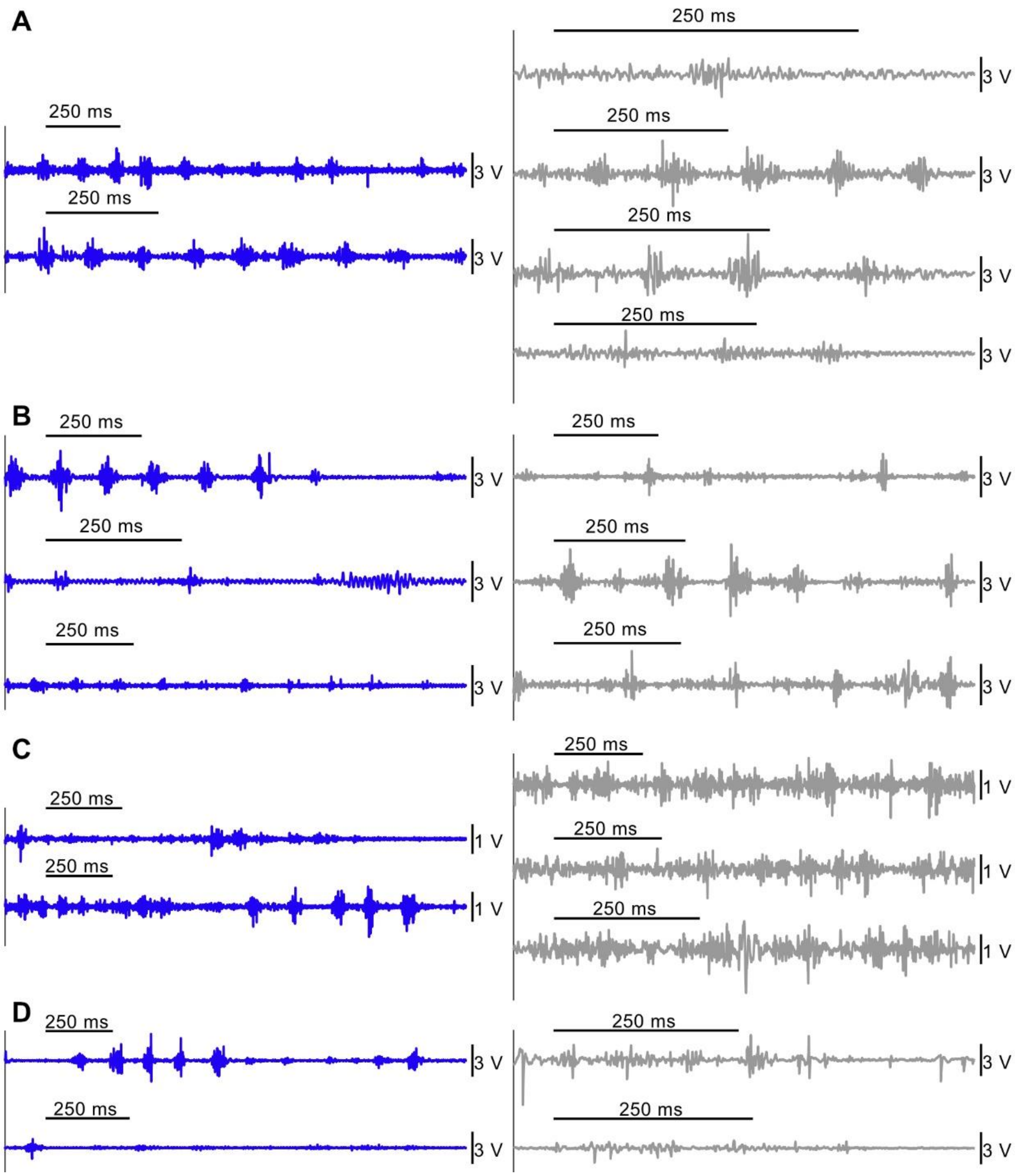

Fig. S1. Muscle activity traces from the adductor of the pectoral fin from 3.0 BD and 0.7 BD for each fish in the EMG set. Each of (A) through (D) is an individual fish. Blue traces in the left column are 3.0 BD trials. Grey traces in the right column are 0.7 BD trials. Each trace is a trial. (A) and (B) were quantified with onsets and offsets in 0.7 BD and therefore included in the duty factor and RIA datasets. (C) and (D) do not show consistent, discrete bursts in 0.7 BD, were not quantified using onsets and offsets at this depth, and thus were not included in the duty factor and RIA datasets at this depth.

Journal of Experimental Biology • Supplementary information

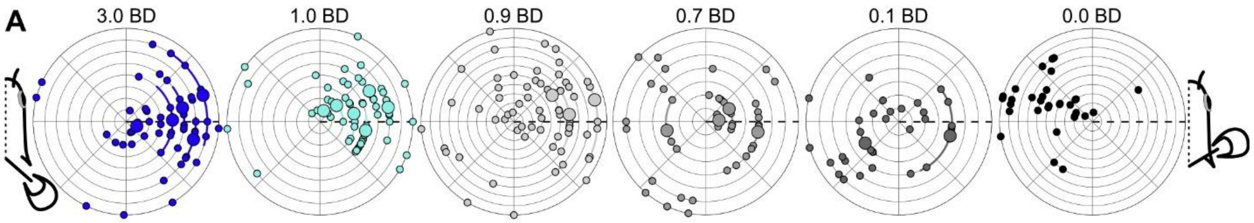

Fig. S2. Pectoral fin timing changes across water depths. Left pectoral fin timing; start of right fin adduction at 0 degrees – dashed line; start of right fin abduction at 180 degrees (N=5 individuals). Each ring is a trial; small points are individual observations of the start of left fin adduction, and large points are angular means for each trial. Angular means and their 95% confidence interval (lines) are presented only when data is directional (Rayleigh's test p-value < 0.05) within a trial.

Table S1. Linear mixed effects model forms.

Dependant Variable	Fixed Effects	Random Effects	Variance Structure
**Locomotion Speed**	Depth	~1|Fish	n/a
**Curvature Coefficient**	Depth	~1|Fish	~1|Depth
**Swing Distance**	Depth*Site	~1|Fish	~1|Site
**Wave Frequency**	Speed*Depth+Site	~1|Fish	~1|Depth
**Fin Frequency**	Speed*Depth	~1|Fish	~1|Depth
**Fin RoM**	Depth	~1|Fish	~1|Depth
**Nose Elevation**	Depth	~1|Fish	n/a
**Body EMG Duty Factor**	Depth+Site	~1|Fish	n/a
**Pectoral Fin EMG Duty Factor**	Depth	~1|Fish	~1|Depth
**Body RIA**	Depth*Site	~1|Fish	~1|Site
**Pectoral Fin RIA**	Speed+Depth	~1|Fish	n/a

Depth is water depth, Site is position on the body, Speed is locomotion speed, Fish is fish identity. A plus sign indicates no interaction between factors; an asterisk indicates an interaction between factors. ~1| before a random effect indicates that the intercept can vary randomly. ~1| in the variance structure indicates that the variance estimate for that effect is allowed to vary.
“n/a” is placed where no variance structure was needed.

Journal of Experimental Biology • Supplementary information

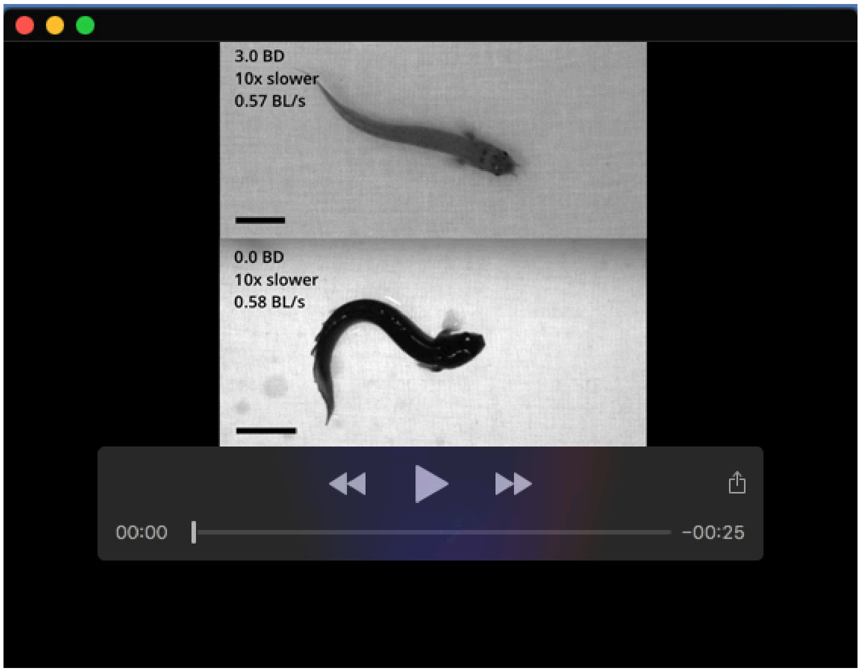

Movie 1. Swimming (top panel) and walking (bottom panel) from two different fish (kinematics set) at a similar speed. Water depth is shown in the top left corner of each panel. Scale bar is 20 mm. BD – body depths, BL – fish total body length.

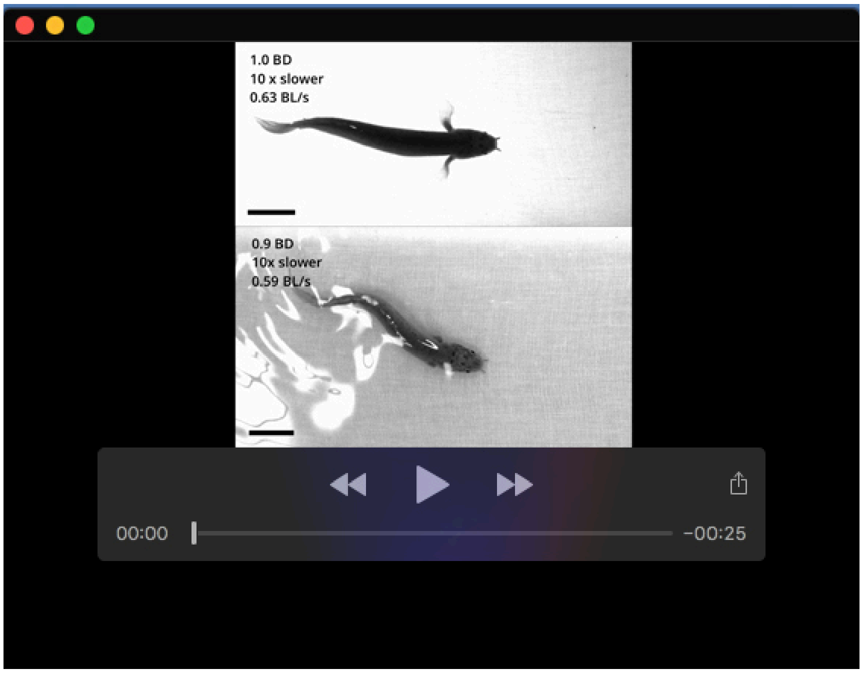

Movie 2. Locomotion (same fish at a similar speed; kinematics set) in intermediate water depths showing changes in body movement. Water depth is shown in the top left corner of each panel. Scale bar is 20 mm. BD – body depths, BL – fish total body length.

Journal of Experimental Biology • Supplementary information

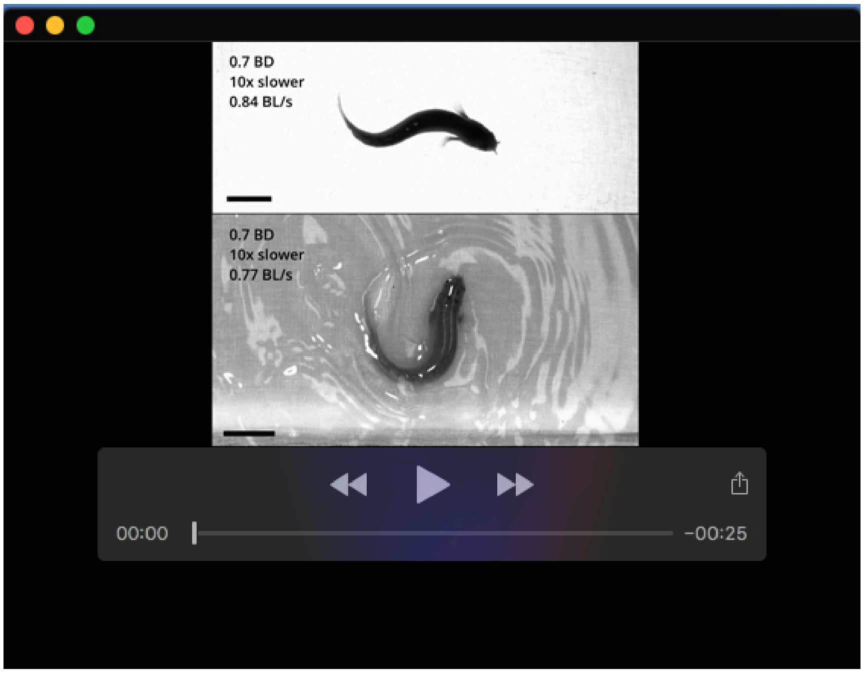

Movie 3. Locomotion (different fish at a similar speed; kinematics set) in 0.7 BD with discrete differences in fin coordination between trials. Water depth is shown in the top left corner of each panel. Scale bar is 20 mm. BD – body depths, BL – fish total body length.

Journal of Experimental Biology • Supplementary information
